# Supplementary material for: Regulation of endocrine cell alternative splicing revealed by single-cell RNA sequencing in type 2 diabetes pathogenesis
Source: Commun Biol. 2024 Jun 27;7:778. doi: 10.1038/s42003-024-06475-0 (PMC11211498; doi:10.1038/s42003-024-06475-0)
Supplement: Supplementary file 2 — Supplementary Information [file 42003_2024_6475_MOESM2_ESM.pdf]

Main Manuscript for

Regulation of endocrine cell alternative splicing revealed by single-cell RNA sequencing in type 2 diabetes pathogenesis

This PDF file includes:

Supporting text
Figures S1 to S20
Tables S1 and S2
Description for Supplementary Data

Other supporting materials for this manuscript include the following:

Supplementary Data

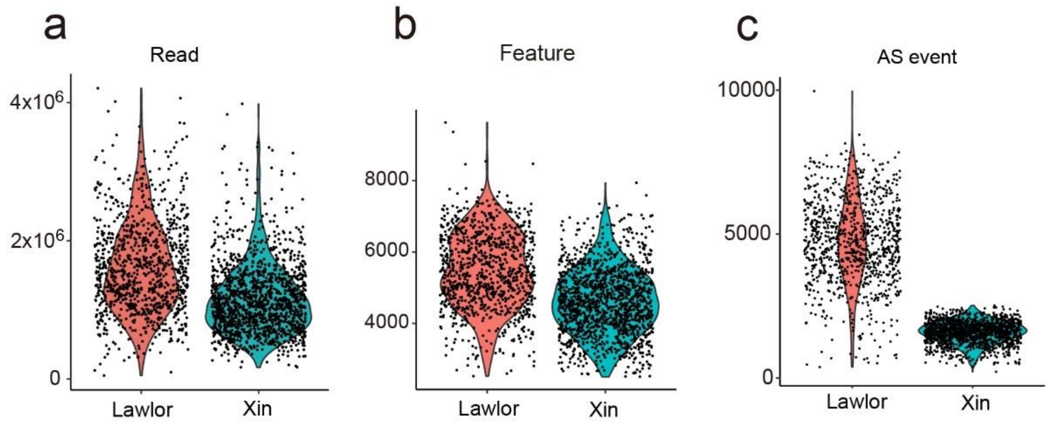

Fig. S1. Basic characteristics of the Lawlor and the Xin datasets

a-c Violin plots of the reads, gene numbers and AS events of the Lawlor and the Xin datasets.

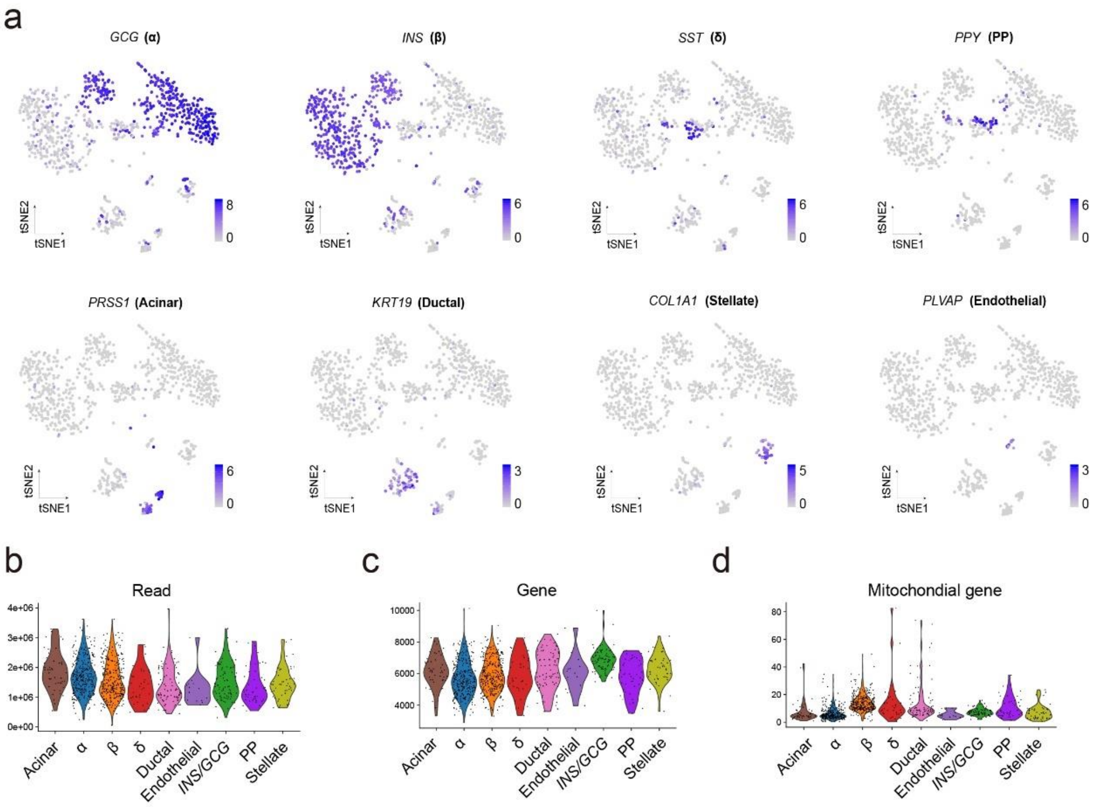

Fig. S2. The cell type annotation and basic characteristics of the Lawlor dataset

a Selected marker gene expression in islet cell subpopulations. **b-d** Violin plots of the reads, gene numbers and mitochondrial gene expression percentage of each cell type from the Lawlor dataset.

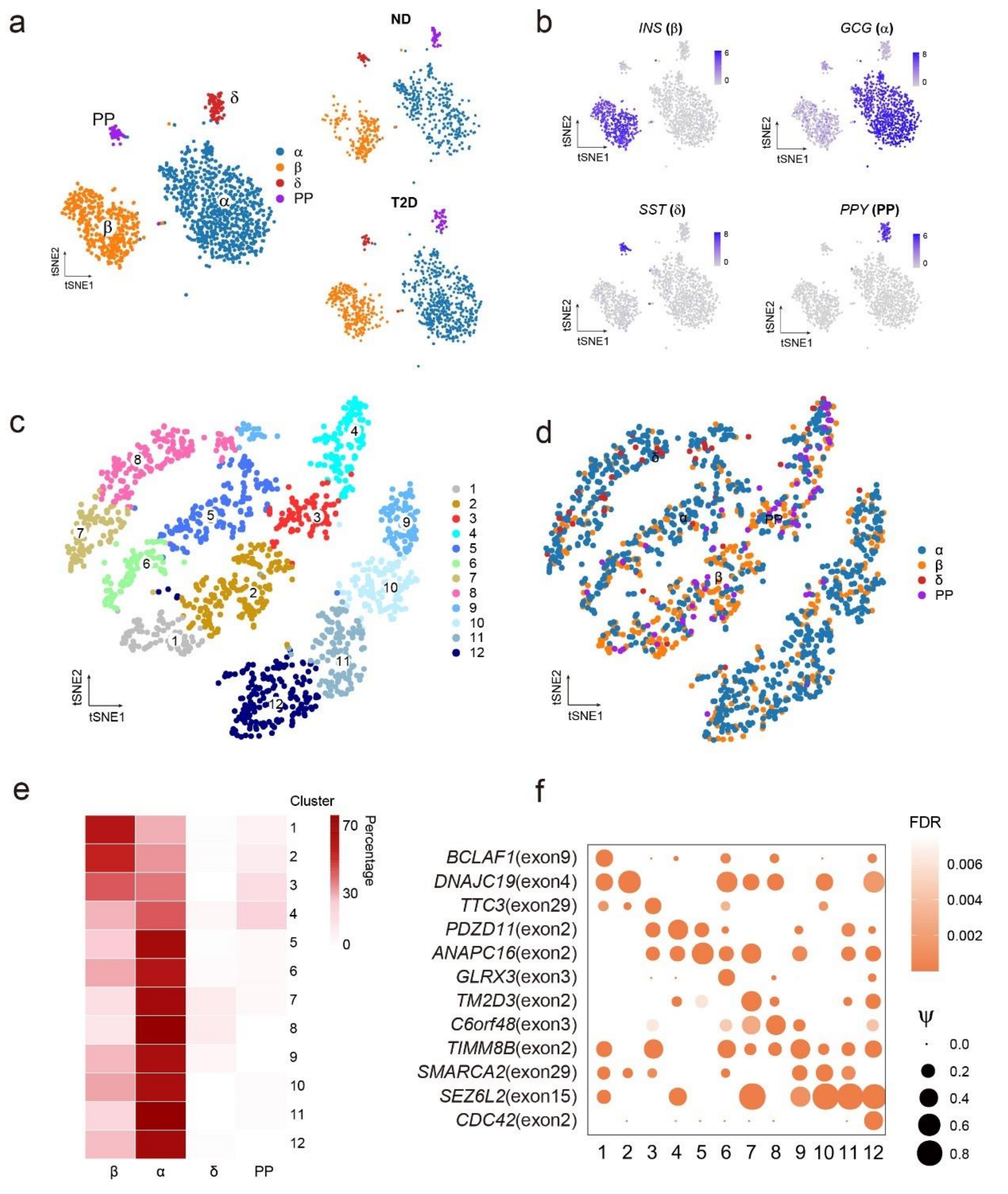

Fig. S3. Single-cell splicing profiles reveal endocrine cell types in the Xin dataset

a t-SNE plots of islet cells from the Xin dataset. **b** Endocrine cell marker gene expression in islet cell subpopulations. **c, d** t-SNE plot of 1,474 endocrine cells from the Xin dataset. Cells are colored by cluster based on the splicing profiles (**c**) and gene expression profiles (**d**). **e** Heatmap showing the overlap between the clusters defined based on the splicing profiles and the cell types defined based on marker gene expression. The color key denotes the percentage of cells in each cluster defined based on the splicing profiles overlapped with the cell types. **f** Bubble plot showing inclusion levels (ψ) of specific splicing exons in each cluster based on splicing profiles. Dot size indicates the ψ value and the color represents the FDR value.

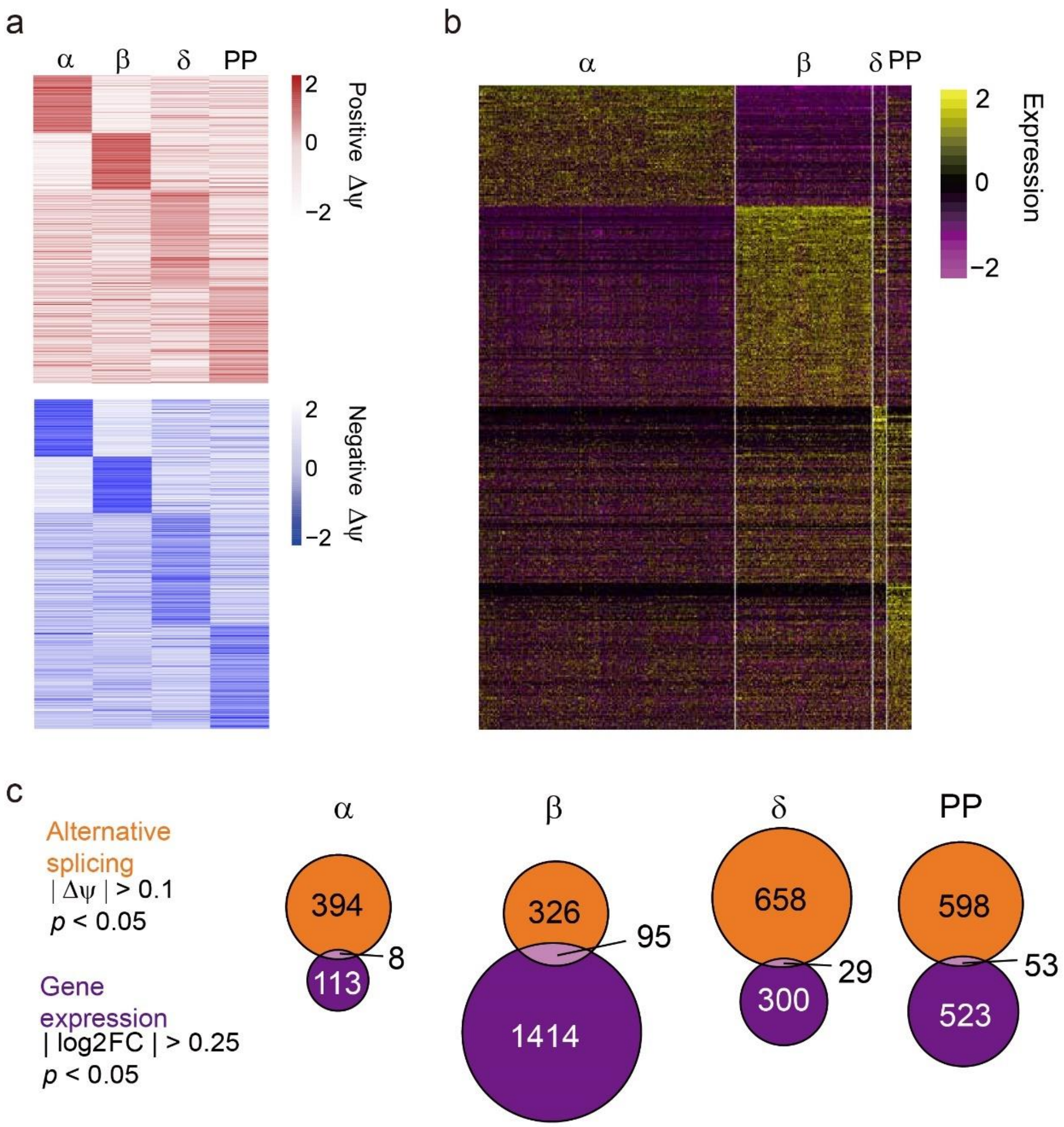

Fig. S4. Cell-type-specific splicing events in endocrine cells from the Xin dataset

a Heatmaps showing relative exon inclusion level (ψ) of cell-type specific exons in each endocrine cell type from the Xin dataset. Red and blue represent significant included ($\Delta\psi > 0.1$ and adjusted p value < 0.05) and skipped ($\Delta\psi < -0.1$ and adjusted p value < 0.05) in each cell type. **b** Heatmap of signature gene expression in endocrine cell types ($\log_2$ FC > 0.25 and adjusted p value < 0.05). Columns denote cells; rows denote genes. Row-scaled expression of the signature genes in each cell type scaled by z-score. **c** Overlapped gene numbers of cell-type-specific exons and cell-type signature genes in α-, β-, δ- and PP cells.

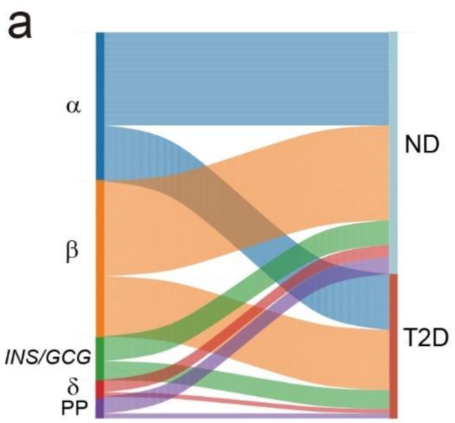

Fig. S5. Endocrine cells from ND and T2D individuals

a Sankey plot showing the cell types defined based on gene expression profiles under ND and T2D conditions from the Lawlor dataset.

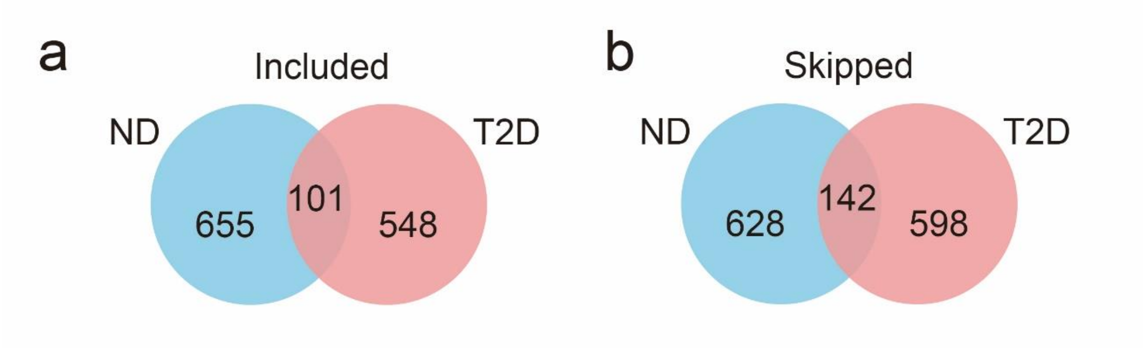

Fig. S6. Included and skipped cell-type-specific exons shared in ND and T2D β-cells

a, b The differential included (**a**, $\Delta\psi > 0.1$ and adjusted *p* value < 0.05) and skipped (**b**, $\Delta\psi < 0.1$ and adjusted *p* value < 0.05) cell-type-specific exons shared in ND and T2D β-cells from the Lawlor dataset.

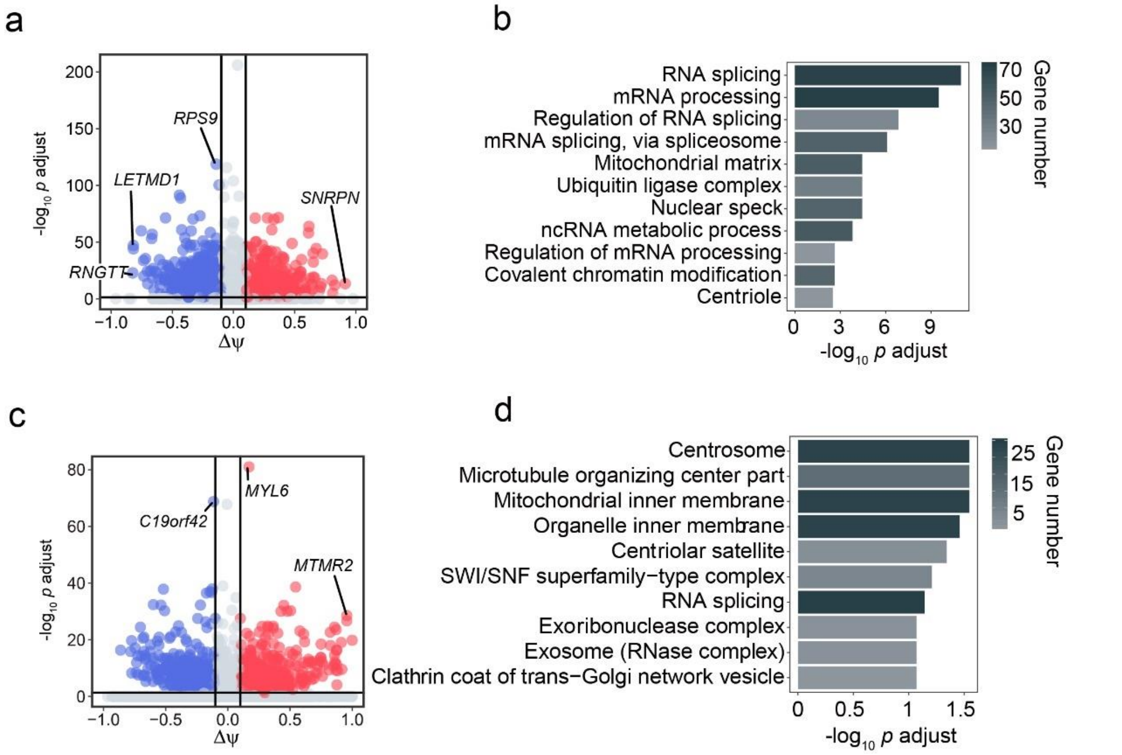

Fig. S7. Differential splicing events in α-cells and *INS*/*GCG*-cells between ND and T2D of the Lawlor dataset

a, c Volcano plot showing differential splicing exons ($|\Delta\psi| > 0.1$ and adjusted *p* value < 0.05) between ND and T2D from α-cells (**a**) and *INS/GCG*-cells (**c**). Red indicates exon included ($\Delta\psi > 0.1$) and blue for exon skipped ($\Delta\psi < -0.1$) in T2D α -cells (**a**) and *INS/GCG*-cells (**c**). **b, d** GO analysis of differential splicing genes between ND and T2D from α-cells (**b**) and *INS/GCG*-cells (**d**).

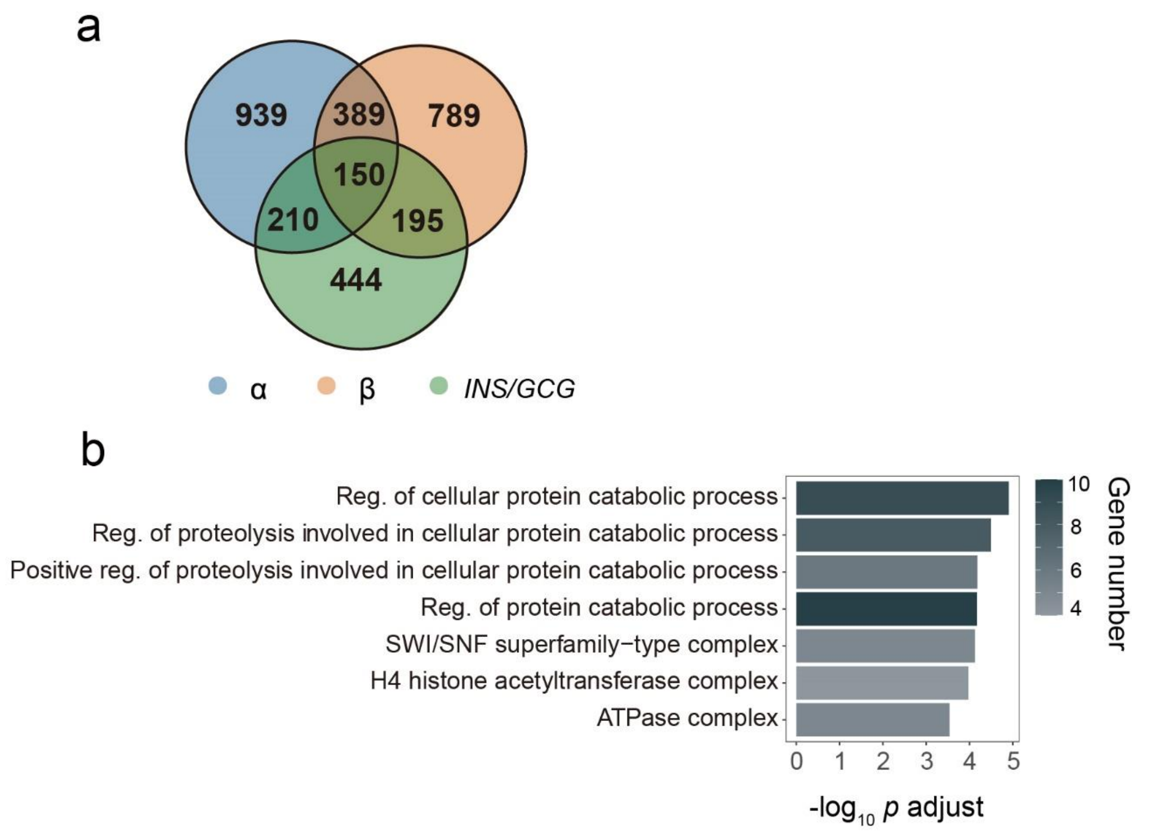

Fig. S8. Differential splicing events in α-, β-, and *INS*/*GCG*-cells between ND and T2D in the Lawlor dataset

a Differential splicing exons ($|\Delta\psi| > 0.1$ and adjusted p value < 0.05) of T2D verses ND in α-, β-, *INS/GCG*- and cells in the Lawlor dataset. **b** GO analysis of the 150 overlapped splicing genes of three cell types in (**a**).

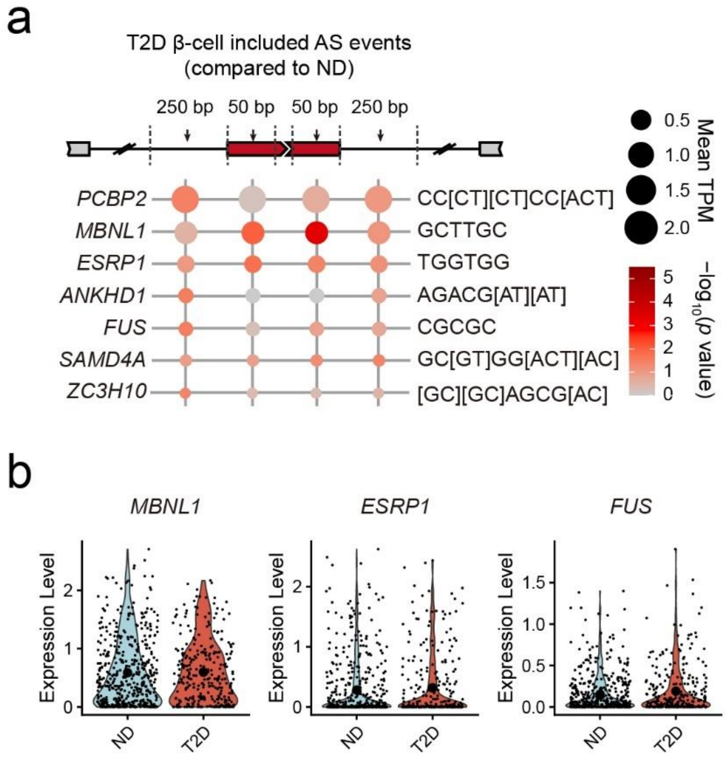

Fig. S9. The predicted splicing factors of including AS events of T2D β-cells

a Bubble plot showing the predicted splicing factors from unbiased motif analysis of included AS events of T2D β-cells compared to ND β-cells in the Lawlor dataset. Motif sequences of corresponding splicing factors were labeled on the right. The dot color represents the smallest *p* value in each enriched region, while the dot size indicates the median expression level of the splicing factors in T2D β-cells. TPM, transcripts per million reads. **b** Violin plots showing the predicted splicing factor expression in ND and T2D β-cells.

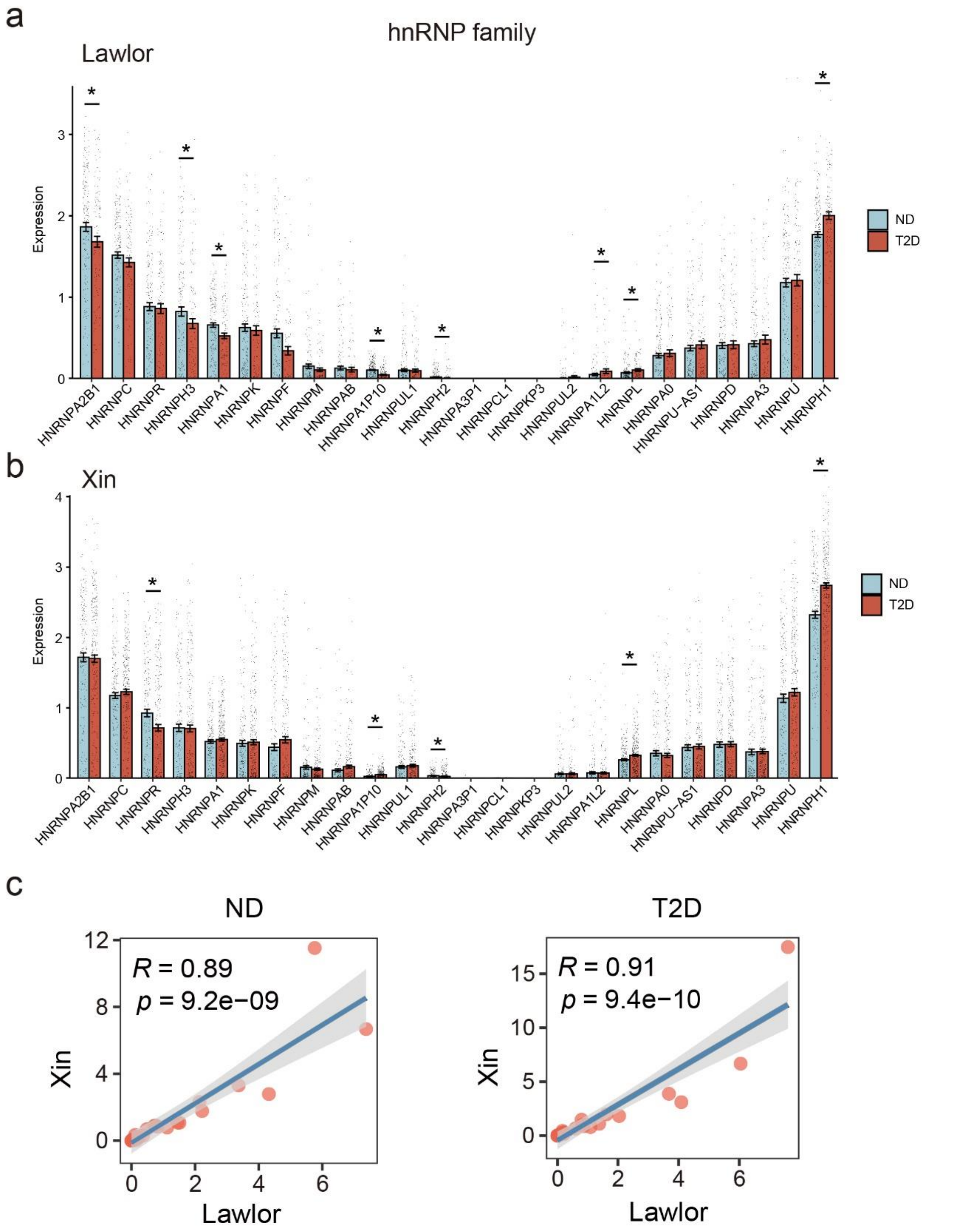

Fig. S10. The gene expression of the hnRNPs in β-cells

a, b Barplots representing the expression of hnRNP family in T2D compared to ND β-cells in the Lawlor dataset (**a**) and the Xin dataset (**b**). **c** Scatter plot showing the correlation of hnRNP family gene expression between the Lawlor dataset and the Xin dataset in ND and T2D. A two-sample KS test was performed to assess statistical significance (**a, b**), * p <0.05.

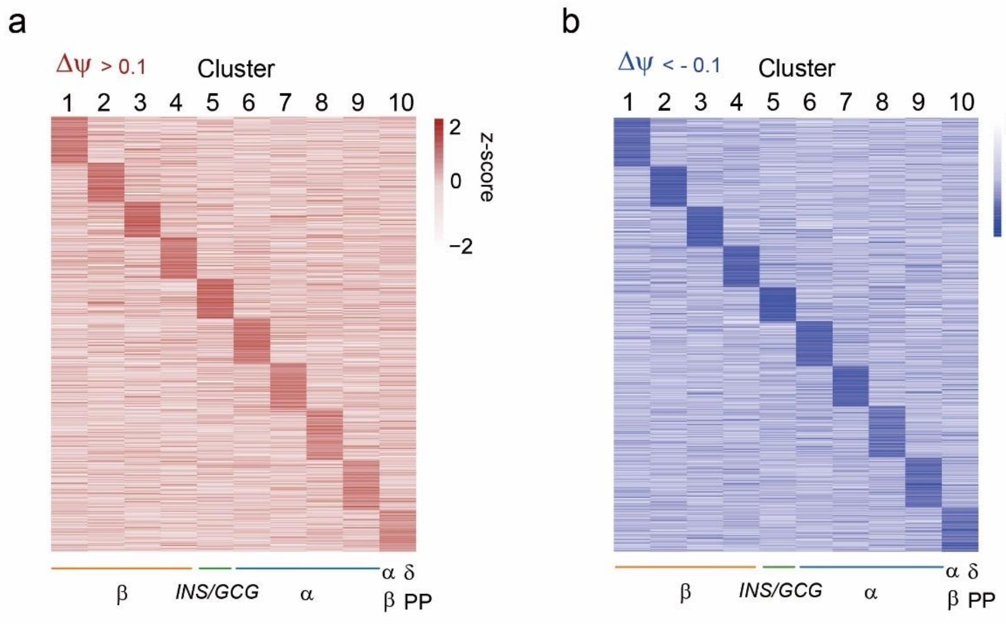

Fig. S11. AS-cluster-specific splicing events in endocrine cells from the Lawlor dataset

a, b Heatmap showing relative exon inclusion level (ψ) of AS cluster specific exons in endocrine cells from the Lawlor dataset. Red represents significant included ($\Delta\psi > 0.1$ and adjusted p value < 0.05). It was detected with 764 (cluster 1), 671 (cluster 2), 699 (cluster 3), 867 (cluster 4), 834 (cluster 5), 773 (cluster 6), 789 (cluster 7), 844 (cluster 8), 790 (cluster 9) and 546 (cluster 10) differential included exons in 558, 522, 533, 631, 640, 581, 596, 618, 573, 412 genes (**a**), and 814 (cluster 1), 699 (cluster 2), 746 (cluster 3), 848 (cluster 4), 729 (cluster 5), 797 (cluster 6), 678 (cluster 7), 839 (cluster 8), 793 (cluster 9) and 582 (cluster 10) differential skipped exons in 609, 527, 576, 650, 587, 602, 523, 628, 595, 415 genes (**b**).

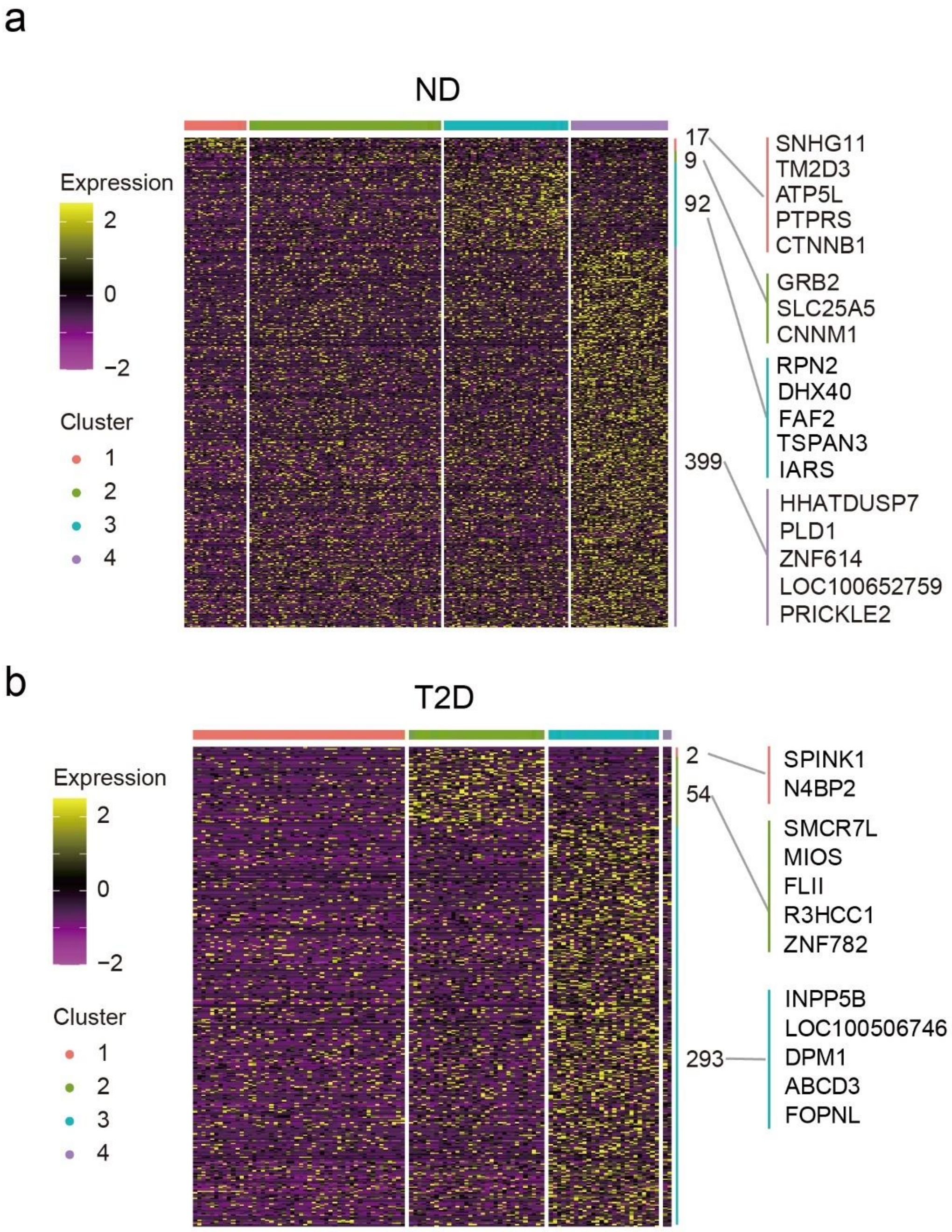

Fig. S12. Signature gene expression of clusters 1 to 4 in the Lawlor dataset

a, b Heatmap of signature gene expression in ND (**a**) or T2D (**b**) β-cells.

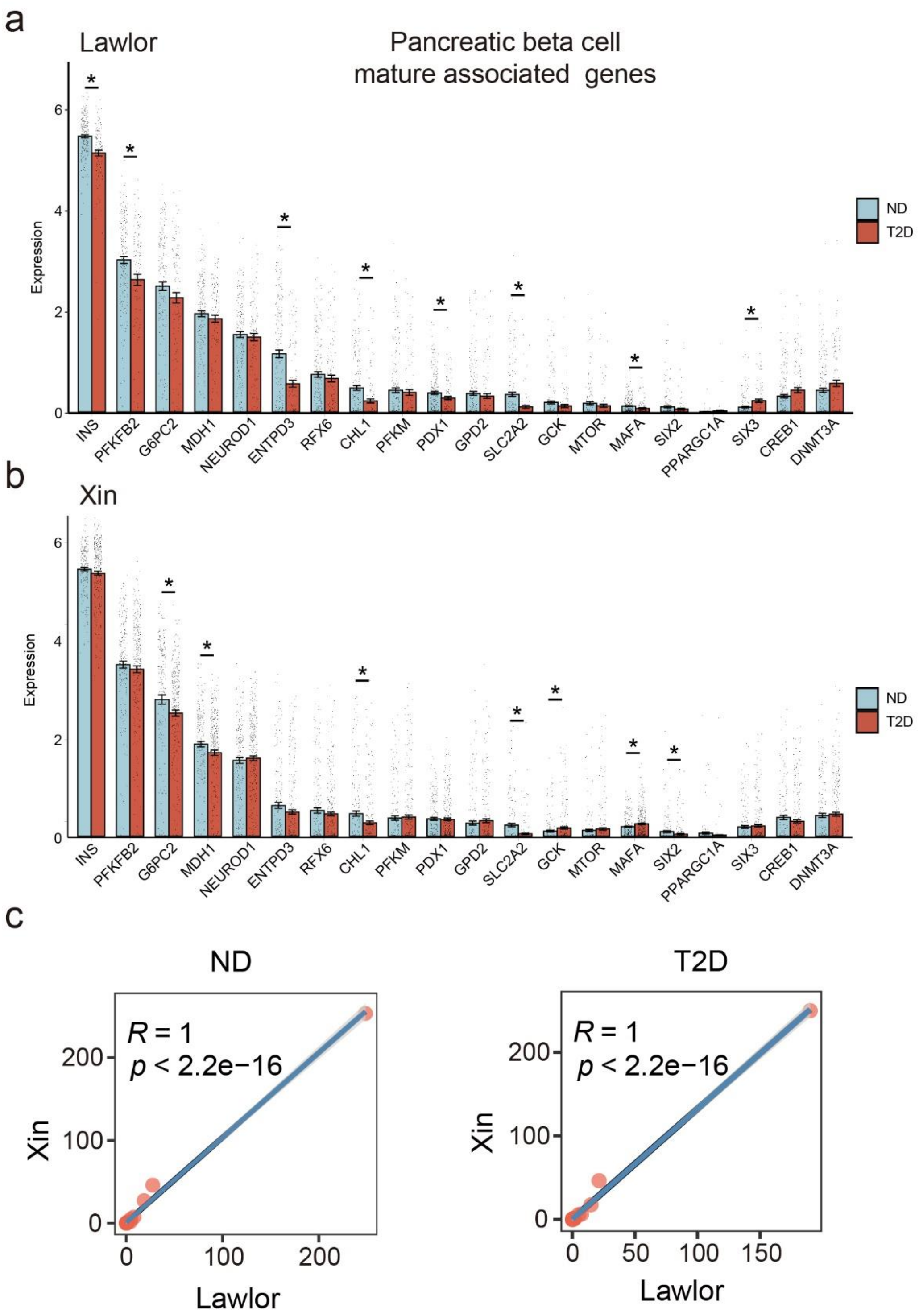

Fig. S13. Gene expression of the β-cell mature markers

a, b Bar plots representing the expression of mature genes in T2D compared to ND β-cells in the Lawlor dataset (**a**) and the Xin dataset (**b**). **c** Scatter plot showing the correlation of the β-cell mature gene expression between the Lawlor dataset and the Xin dataset in ND and T2D. A two-sample KS test was performed to assess statistical significance (**a, b**), * p <0.05.

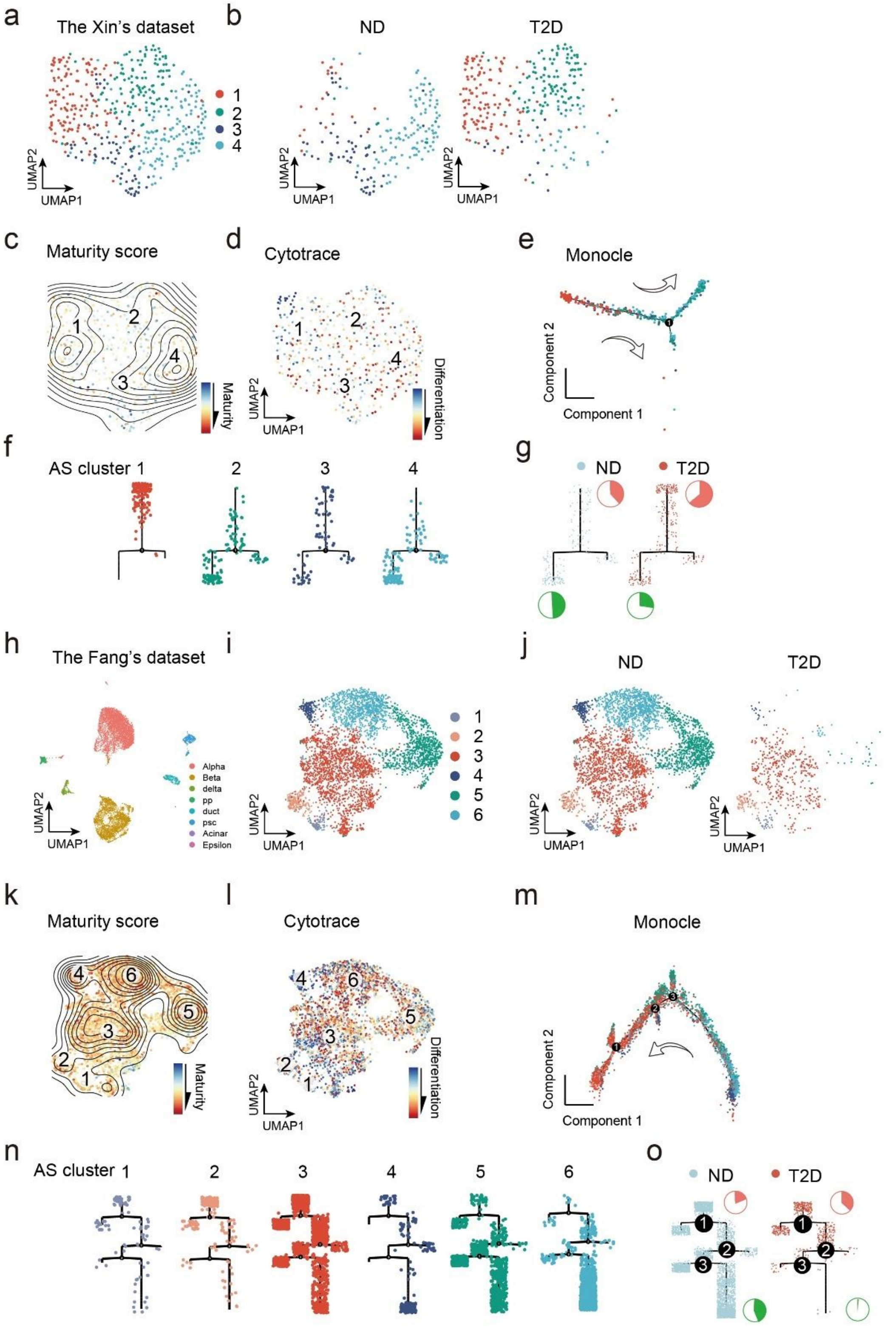

a
The Xin's dataset
b
ND
T2D
c
Maturity score
d
Cytotrace
e
Monocle
Component 2
Component 1
f
AS cluster 1
g
ND
T2D
h
The Fang's dataset
Alpha
Beta
delta
pp
duct
psc
Acinar
Epsilon
i
j
ND
T2D
k
Maturity score
l
Cytotrace
m
Monocle
Component 2
Component 1
n
AS cluster
o
ND
T2D
UMAP1
UMAP2
Maturity
Differentiation

Fig. S14. Mature β-cells decrease in T2D of the Xin dataset and the Fang dataset

a, b UMAP plot of ND and T2D β-cells from the Xin dataset. Cells are colored by clusters based on the gene expression profiles. **c** UMAP plot showing maturity scores of clusters 1 to 4 from β-cells overlaid with a contour map corresponding to maturity scores. The color indicates maturity scores. **d** CytoTRACE UMAP plot of clusters 1 to 4. The color indicates the level of differentiation from low (blue) to high (red). **e** Monocle 2 pseudotime trajectory of clusters 1 to 4 cells. Arrows indicate the trajectory of pseudotime pathway. **f, g** Pseudotime trajectory of clusters 1 to 4 (**f**), ND and T2D (**g**), respectively. **h** UMAP plot of 27,995 islet cells from the Fang dataset. Cells are colored by annotated cell types. **i, j** UMAP plot of ND and T2D β-cells from the Fang dataset. Cells are colored by clusters based on the gene expression profiles. **k** UMAP plot showing maturity scores of clusters 1 to 6 from β-cells overlaid with a contour map corresponding to maturity scores. The color indicates maturity scores. **l** CytoTRACE UMAP plot of clusters 1 to 6. The color indicates the level of differentiation from low (blue) to high (red). **m** Monocle 2 pseudo-time trajectory of clusters 1 to 6 cells. Arrows indicate the trajectory of pseudotime pathway. **n, o** Pseudo-time trajectory of clusters 1 to 6 (**n**), ND and T2D (**o**), respectively.

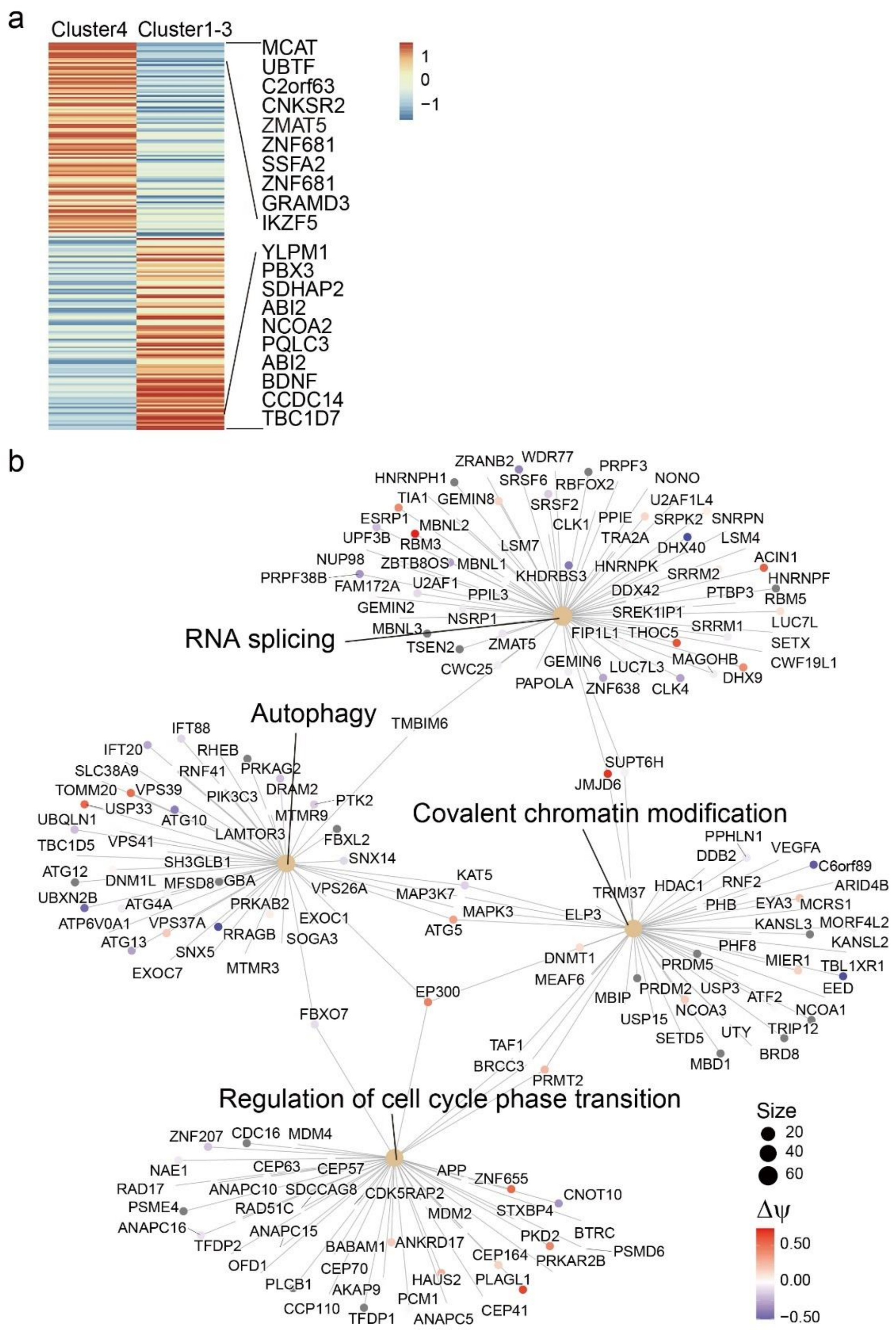

Fig. S15. Differential splicing events of cluster 4 compared to clusters 1 to 3 in the Lawlor dataset

a Heatmap showing included ($\Delta\psi$ > 0.1 and adjusted *p* value < 0.05) or skipped ($\Delta\psi$ < -0.1 and adjusted *p* value < 0.05) splicing genes of cluster 4 compared to clusters 1 to 3. **b** Cnetplot of enriched pathways in (Fig. 6l).

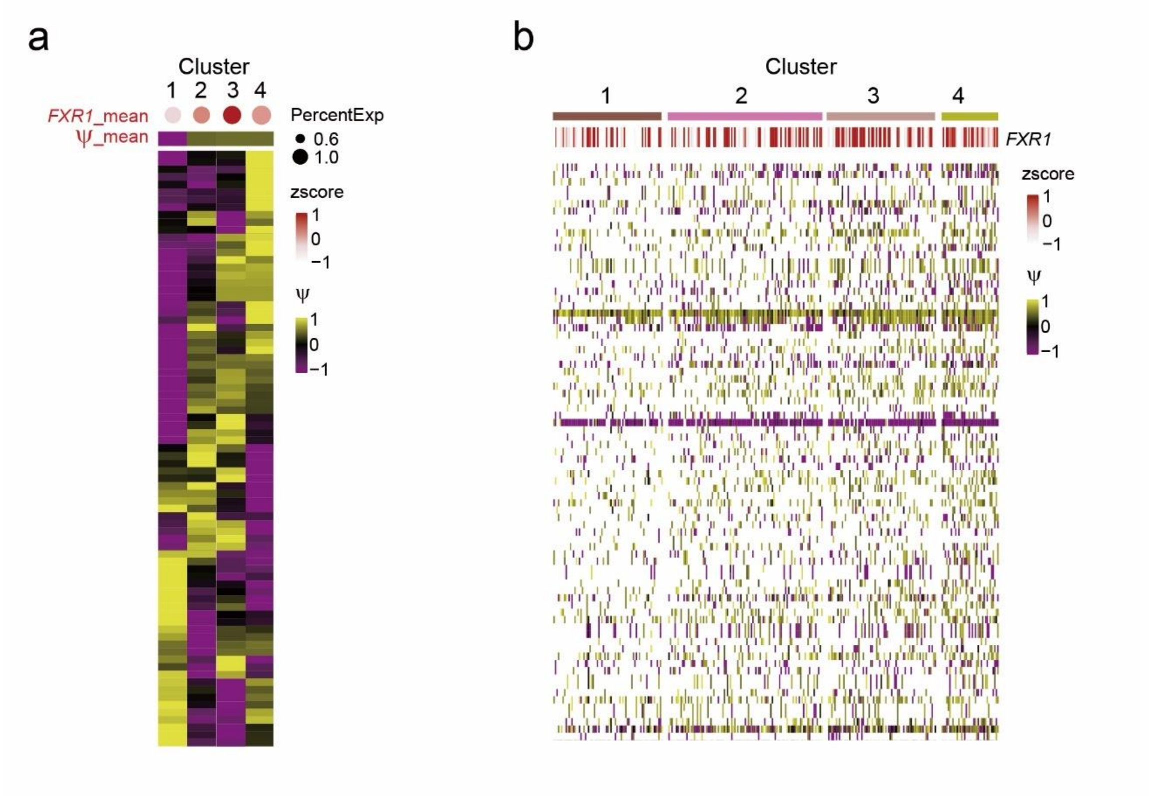

Fig. S16. *FXR1* expression and the inclusion levels of its potential targets across β-cells

a, b Heatmaps showing *FXR1* expression and the inclusion level of 79 potentially targeted exons (in 70 genes) across β-cells at the single-cell level (**a**) and across subsets (**b**). Dot size indicates the percentage of *FXR1* expression in each cluster in (**a**), and the color is the z-scores of *FXR1* average expression (upper) and z-scores of ψ (down) in (**a, b**).

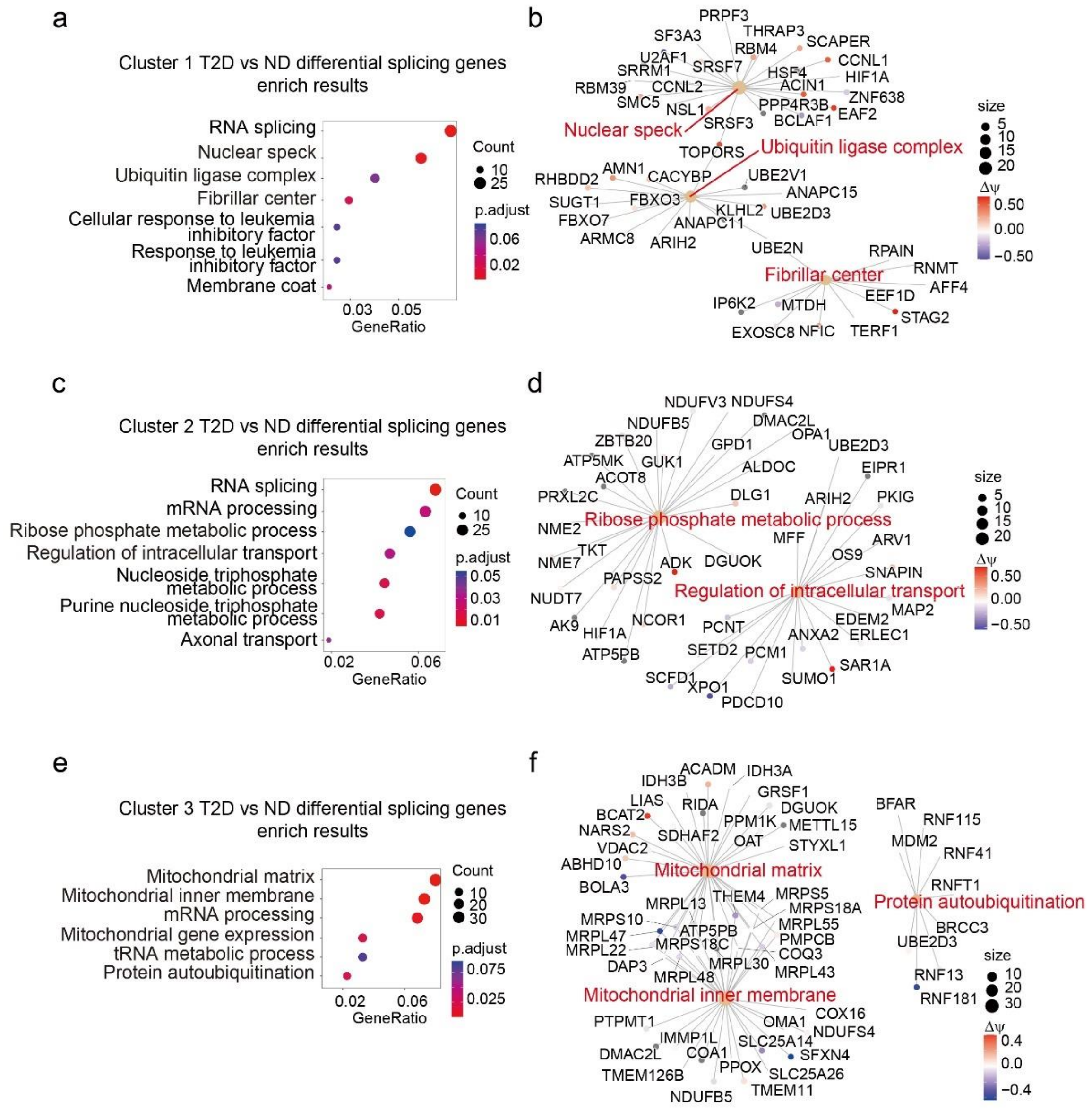

Fig. S17. Differential splicing events of T2D cells compared to ND cells in clusters 1 to 3

a, c, e GO analysis of specific splicing genes (|Δψ| > 0.1 and adjusted *p* value < 0.05) of T2D cells compared to ND cells in clusters 1 to 3 of the Lawlor dataset. **b, d, f** Cnetplot of enriched pathways in (**a, c, e**), respectively.

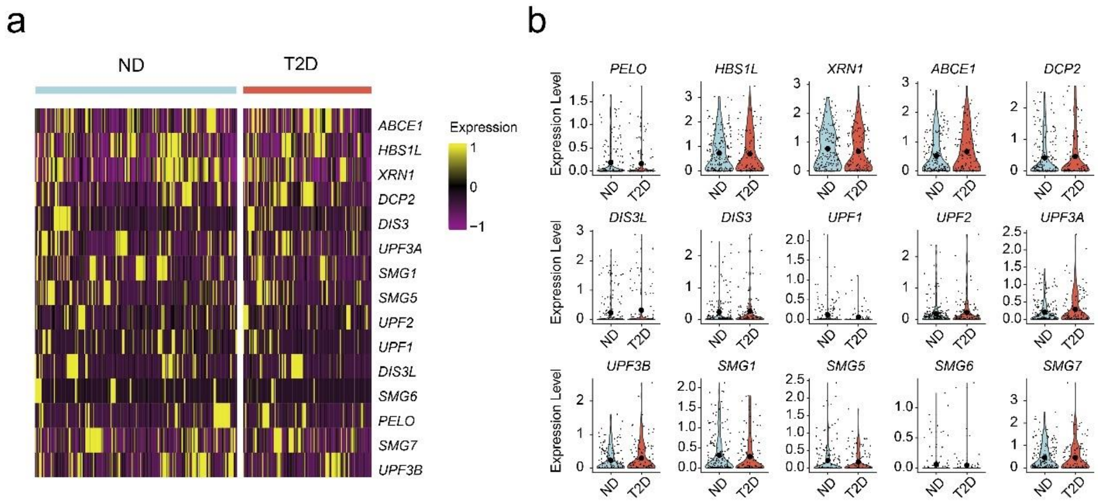

Fig. S18. NMD gene expression in ND and T2D β-cells

a, b Heatmap (**a**) and violin plots (**b**) showing NMD gene expression in ND and T2D β-cells. A two-sample KS test was performed to assess statistically significant (**b**), * p <0.05.

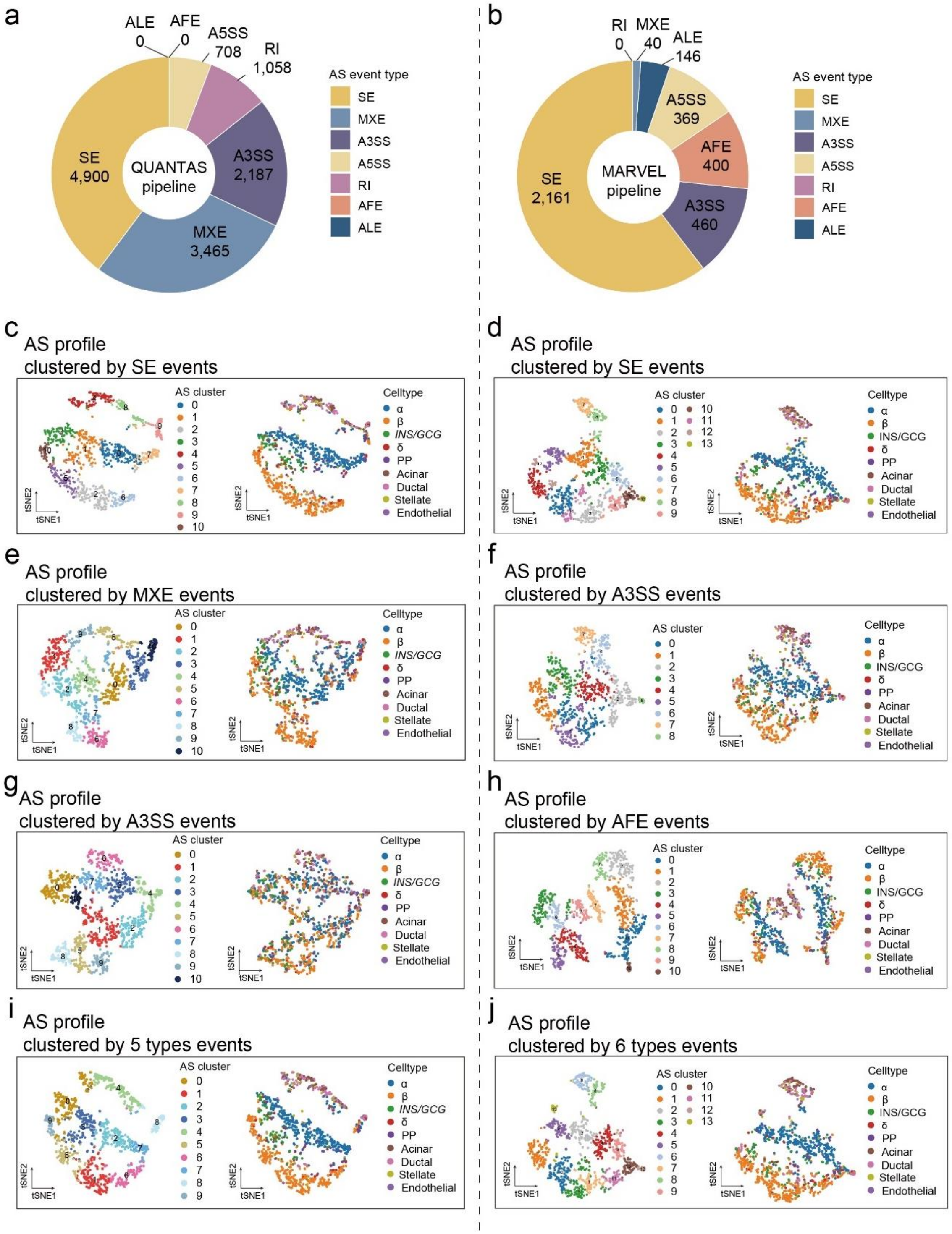

Fig. S19. AS events quantification by QUANTAS pipeline and MARVEL pipeline

a, b Seven AS event types quantified by QUANTAS (**a**) and MARVEL (**b**) from the Lawlor dataset. **c-j** t-SNE plot clustered by individual AS event types (**c-h**) and all seven AS event types (**i, j**). Cells are colored by cluster based on the splicing profiles (left) and gene expression profiles (right) in each panel.

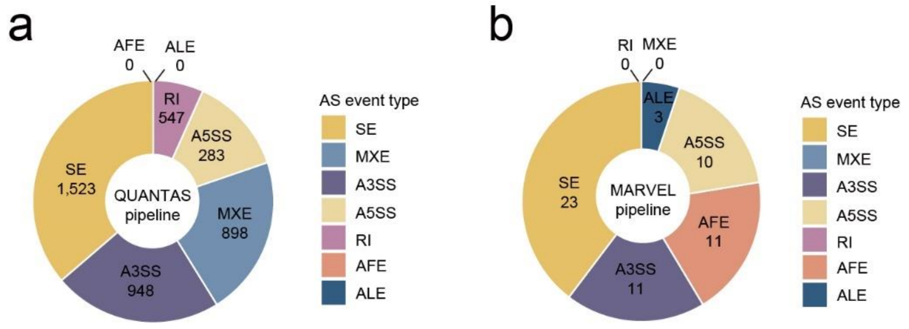

Fig. S20. Differential splicing events in β-cells between ND and T2D quantified by Quantas and MARVEL

a Pie chart showing differential splicing exons ($|\Delta\psi| > 0.1$ and adjusted *p* value < 0.05) detected by Quantas pipeline in the Lawlor dataset. **b** Pie chart showing differential splicing exons (adjusted *p* value < 0.1 and outlier = FALSE) detected by MARVEL pipeline.

Table S1. Human pancreas single cell RNA-seq datasets.

Author	*Method*	*Cell num*	*Count (per cell)*	*Gene number (per cell)*	*Status*	*scRNA-seq Platforms*	*Sequence read length*	*Deposited Data*	*Single/paired-end*
**SMART seq**									
Lawlor N; Genome Res.; 2017 [39]	full transcript	972	1.61 million	5,468	5 ND, 3 T2D	Fluidigm C1	75 bp	GSE86473	single
Xin YR; Cell Metab.; 2016 [38]	full transcript	1474	1.15 million	4,613	12 ND, 6 T2D	Fluidigm C1	50 bp	GSE81608	single
Segerstolpe A; Cell Metab.; 2016 [36]	full transcript	2166	0.48 million	6,325	6 ND, 4 T2D	Smart-seq2	43 bp	E-MTAB-5061	single
Wang YJ; Diabetes; 2016 [37]	full transcript	316	/	/	6 Adult (3 ND, 1 T1D, 2 T2D)	Fluidigm C1	100 bp	GSE83139	single
Avrahami D; Mol. Metab.; 2020 [34]	full transcript	407	2.26 million	6,067	14 Adult (4 ND, 10 T2D)	Fluidigm C1	100 bp	GSE154126	single
**Drop seq**									
Fang Z; Cell Report; 2019 [35]	5'/3' end	27995	1054	510	6 ND, 3 T2D	Drop-Seq	50 bp	GSE101207	paired
Baron M; Cell Syst; 2016 [40]	5'/3' end	8569	5828	1887	3 ND, 1 T2D	inDrop	/	GSE84133	paired

Table S2. The predicted splicing factors from unbiased motif analysis. Related to figure 6K.

RBP	*Motif*	*Smallest p in upstream intron*	*Smallest p in target exon 5'*	*Smallest p in target exon 3'*	*Smallest p in downstream intron*	*Enriched in*
**HNRNPA2B1**	[AGT]TAGG G[AT]	0.074001	0.507529	0.031072	0.018981	Clusters 1-3
**HNRNPA1**	[AGT]TAGG G[AT]	0.074001	0.507529	0.031072	0.018981	Clusters 1-3
**HNRNPA1L2**	[AGT]TAGG G[AT]	0.074001	0.507529	0.031072	0.018981	Clusters 1-3
**MSI1**	TAGT[AT][A G]G	0.014798	0.507529	0.210155	0.116562	Clusters 1-3
**SNRPA**	[AT]TGCAC[AG]	0.005045	0.024039	0.190358	0.053543	Clusters 1-3
**FXR1**	A[CT]GAC[A G]	0.013175	0.32299	0.080941	0.250872	Clusters 1-3
**HNRNPK**	CCA[AT][AC]CC	0.003181	0.014542	0.053543	0.020043	Clusters 1-3
**MBNL1**	GCTTGC	0.085937	0.036938	0.00899	0.232394	Clusters 1-3
**SRSF10**	AGAGA[AC G][AC]	0.008782	0.093361	0.096747	0.134886	Clusters 1-3
**CNOT4**	GACAGA	0.018981	0.54885	0.67371	0.063522	Clusters 1-3
**ZC3H10**	[GC][GC]AG CG[AC]	0.031072	0.023711	0.225425	0.196385	Clusters 1-3
**YBX2**	AACA[AT]C[AGT]	0.005589	0.305041	0.218944	0.014542	Clusters 1-3
**QKI**	ACTAAC[AC G]	0.031072	0.196385	0.031072	0.098982	Clusters 1-3
**ANKHD1**	AGACG[AT][AT]	0.507529	0.031072	0.196385	0.196385	Clusters 1-3
**FMR1**	[GT]GACA[A G]G	0.033501	0.232203	0.160095	0.023711	Clusters 1-3
**PPRC1**	[GC][GC]GC GC[GC]	0.196385	0.196385	0.210155	0.031072	Clusters 1-3
**YBX1**	AACATC	0.036938	0.633147	0.633147	0.085937	Clusters 1-3
**RBM8A**	[AG][CT]GC GC[CGT]	0.065678	0.611244	0.283523	0.031072	Clusters 1-3
**SRSF10**	AGAGA[AC G][AC]	0.219402	0.42665	0.292481	0.049835	Cluster 4
**FXR1**	A[CT]GAC[A G]	0.188981	0.0622	0.022515	0.016157	Cluster 4
**FUS**	CGCGC	0.407592	0.649342	0.407592	0.04017	Cluster 4
**MSI1**	TAGT[AT][A G]G	0.118703	0.325509	0.012121	0.066468	Cluster 4
**ANKHD1**	AGACG[AT][AT]	0.230241	0.083432	0.04017	0.134424	Cluster 4
**ZC3H10**	[GC][GC]AG CG[AC]	0.04017	0.203311	0.139026	1	Cluster 4

Supplementary Data (separate file). The source data behind the graphs in the paper.
